# Formate hydrogenlyase is essential for pH homeostasis, maintenance of ATP levels, and CO_2_ provision in stationary-phase *Escherichia coli*

**DOI:** 10.1128/jb.00007-26

**Published:** 2026-05-28

**Authors:** Christopher Erdmann, Liana Vanyan, Karen Trchounian, R. Gary Sawers

**Affiliations:** 1 Institute for Microbiology, Martin Luther University Halle-Wittenberg 9176, Halle (Saale), Germany; 2 Department of Biochemistry, Microbiology and Biotechnology, Faculty of Biology, Yerevan State University 105430, Yerevan, Armenia; 3 Research Institute of Biology, Yerevan State University 105430, Yerevan, Armenia; University of Florida, Gainesville, Florida, USA

**Keywords:** formate, pH homeostasis, formate hydrogenlyase, ATPase, fermentation, hydrogen

## Abstract

Formate hydrogenlyase (FHL) enzymes are evolutionarily related to mitochondrial complex I. Formate-inducible FHL-1 of *Escherichia coli* catalyzes disproportionation of formic acid into H_2_ and CO_2_ during mixed-acid fermentation. In contrast to FHL-2 enzymes found in many microorganisms, including *E. coli*, FHL-1 shares only two of the usual five membrane-domain subunits, perhaps explaining its apparent inability to translocate protons. This raises the question, however, as to the physiological function of the FHL-1 enzyme. Here, we demonstrate that FHL-1 serves to regulate intracellular pH and CO_2_ levels in stationary-phase fermenting cells. Determination of intracellular and extracellular pH values in *E. coli* strains with and without an FHL-1 enzyme revealed an alkalinization (0.5 pH units) of the cytoplasm in cells lacking FHL-1. This increase in pH_i_ correlated with a concomitant threefold decrease in the ATP concentration in these cells, while the parental strain retained both high ATP levels and a pH_i_ of 6.7. The reduction in ATP levels in the mutant correlated with an increase in F_1_F_o_-ATPase activity. Remarkably, ATP levels and pH_i_ in the FHL-1-negative mutant could be restored to near wild-type levels by growth in the presence of bicarbonate. Together, these data indicate that FHL-1 functions to provide CO_2_/bicarbonate for key carboxylation reactions, concomitantly maintaining intracellular pH homeostasis and thereby indirectly optimizing ATP levels by preventing excessive ATP hydrolysis via F_1_F_o_-ATPase in its attempt to neutralize pH_i_. CO_2_ production by cytoplasmically oriented FHL-1 substitutes for a pyruvate decarboxylation reaction, and explains why maintaining formate homeostasis is central to mixed-acid fermentation.

**IMPORTANCE:**

The signature molecule of enterobacterial mixed-acid fermentation, formate, is disproportionated into H_2_ and CO_2_ by formate hydrogenlyase (FHL), an evolutionarily ancient enzyme phylogenetically related to proton-translocating complex I. However, as FHL-1 of *Escherichia coli* lacks the capacity to translocate protons, its physiological role remains unclear. We show that FHL-1 functions to maintain pH homeostasis and intracellular CO_2_/bicarbonate levels in stationary-phase cells. Mutants lacking a functional FHL-1 fail to generate H_2_ and CO_2_, causing activation of F_1_F_o_-ATPase, which hydrolyzes ATP to maintain neutral cytoplasmic pH. The balance between formate synthesis and its intracellular disproportionation is necessary to maintain the supply of bicarbonate for carboxylation reactions, thus ensuring pH homeostasis and ultimately optimal ATP levels, which together aid stationary-phase survival.

## INTRODUCTION

The ability of microorganisms to transform formic acid into H_2_ and CO_2_ was recognized a century and a half ago, and the enzyme that performs this formate hydrogenlyase (FHL) reaction was first described in 1932 (1, 2). FHL complexes share a common evolutionary ancestor with complex I (NADH dehydrogenase) of the mitochondrial respiratory chain (3–6). Despite the fact that the sequence and structural homologies between complex I and FHL (3, 7, 8) suggest that FHL might be involved in energy conservation, current evidence suggests that the well-characterized FHL-1 complex in *Escherichia coli* does not appear to translocate protons (6, 9). This is possibly because the membrane domain of this L-shaped enzyme complex has only two “anchoring” subunits (3, 8, 9), rather than the usual five-subunit membrane domain found in FHL-2 complexes. These latter enzymes share more similarity with complex I enzymes and might be energy conserving (10–12). The cytoplasmically oriented catalytic domains of FHL-1 and FHL-2 are similar and share a formate dehydrogenase subunit, termed Fdh-H, linked via iron-sulfur cluster-containing subunits to a [NiFe]-hydrogenase 3 enzyme (6). The electrons derived from the oxidation of formate are transferred via the iron-sulfur clusters to the hydrogenase, where proton reduction occurs, releasing membrane-permeant H_2_ gas (6, 8). Notably, this set of reactions is fully reversible in FHL-1 (13), another feature shared with complex I. If FHL-1 does not translocate protons, this begs the question—what is the main physiological function of this enzyme?

It has long been recognized that FHL-1 is substrate-induced (2), meaning that it is synthesized only when formate accumulates in the cytoplasm. The genes encoding the structural components of FHL-1 are part of the formate regulon, and expression of the regulon is controlled by the formate-responsive transcription activator, FhlA (14, 15). During mixed-acid fermentation of glucose, formate is generated during the non-oxidative cleavage of pyruvate into acetyl-CoA by pyruvate formate-lyase (PflB) (16) and the subsequent FHL-1 reaction represents the only possibility for the release of CO_2_.

As much as one-third of the carbon derived from glucose is converted to formate by PflB, so that this strong electron donor, which is used by many microorganisms, accumulates to millimolar concentrations in the cytoplasm and in the immediate environment of the bacterium (17). Translocation of formic acid across the cytoplasmic membrane is achieved by the pentameric formate channel/transporter, FocA (17–19). Notably, of the organic acids produced during mixed-acid fermentation, formic acid is the only one that is reimported nearly completely under standard laboratory conditions, and this is also carried out by FocA, whereby formic acid uptake by FocA occurs most efficiently when the pH of the culture medium decreases below 6.5 (20). Moreover, as the *focA* gene is co-transcribed with *pflB* (21) and expression of the operon is anaerobically induced (22), synthesis of FocA and PflB is co-regulated. Consequently, through efficient pyruvate cleavage by PflB (16), controlled bidirectional FocA-dependent translocation of formic acid, formate-induced synthesis of FHL-1, and its disproportionation to H_2_ and CO_2_, *E. coli* balances cytoplasmic formate and CO_2_ levels while simultaneously maintaining intracellular pH homeostasis. Providing sufficient intracellular CO_2_, which is readily converted to bicarbonate by carbonic anhydrase (23), is essential for key carboxylation reactions, especially the first committed step in lipid biosynthesis (24).

A recent seminal study using labeled formate combined with Raman spectroscopy demonstrated that exogenously supplied formate was quantitatively imported and converted to H_2_ and CO_2_ by the FHL-1 complex (25). This disproportionation of exogenously supplied formate occurred in parallel with, but did not affect the kinetics of, efflux and subsequent reimport of endogenously generated formate, suggesting a direct route for exogenous formic acid to FHL-1, possibly via FocA. These studies clearly supported a role for FHL-1 in modulating pH homeostasis (25). Results of an earlier study showed that hydrogenases, especially hydrogenase 3, are important for the acid-resistance phenotype of anaerobically grown *E. coli* (26), and the authors suggested that H_2_ production and thus FHL-1 activity are important in allowing the bacterium to survive short-term extremes of acid pH, e.g., during transient passage through the stomach. Finally, a study on *atp* mutants of *Salmonella enterica* Typhimurium, which lacked a fully functional proton-translocating F_1_F_o_-ATPase, showed defective H_2_ production, interpreted at the time as revealing a link between proton-translocation capability and redox activity of fermenting cells (27). In this current study, we show that FHL-1 is indeed required for the maintenance of cellular pH homeostasis; however, this proves to be related to its ability to produce CO_2_. The findings of the study also help explain the link between FHL-1, the activity of F_1_F_o_-ATPase, cellular carbon and energy metabolism, and stationary-phase survival during mixed-acid fermentation.

## RESULTS

### Early transition into the stationary phase of *E. coli* lacking a functional FHL-1

**Fig 1:**
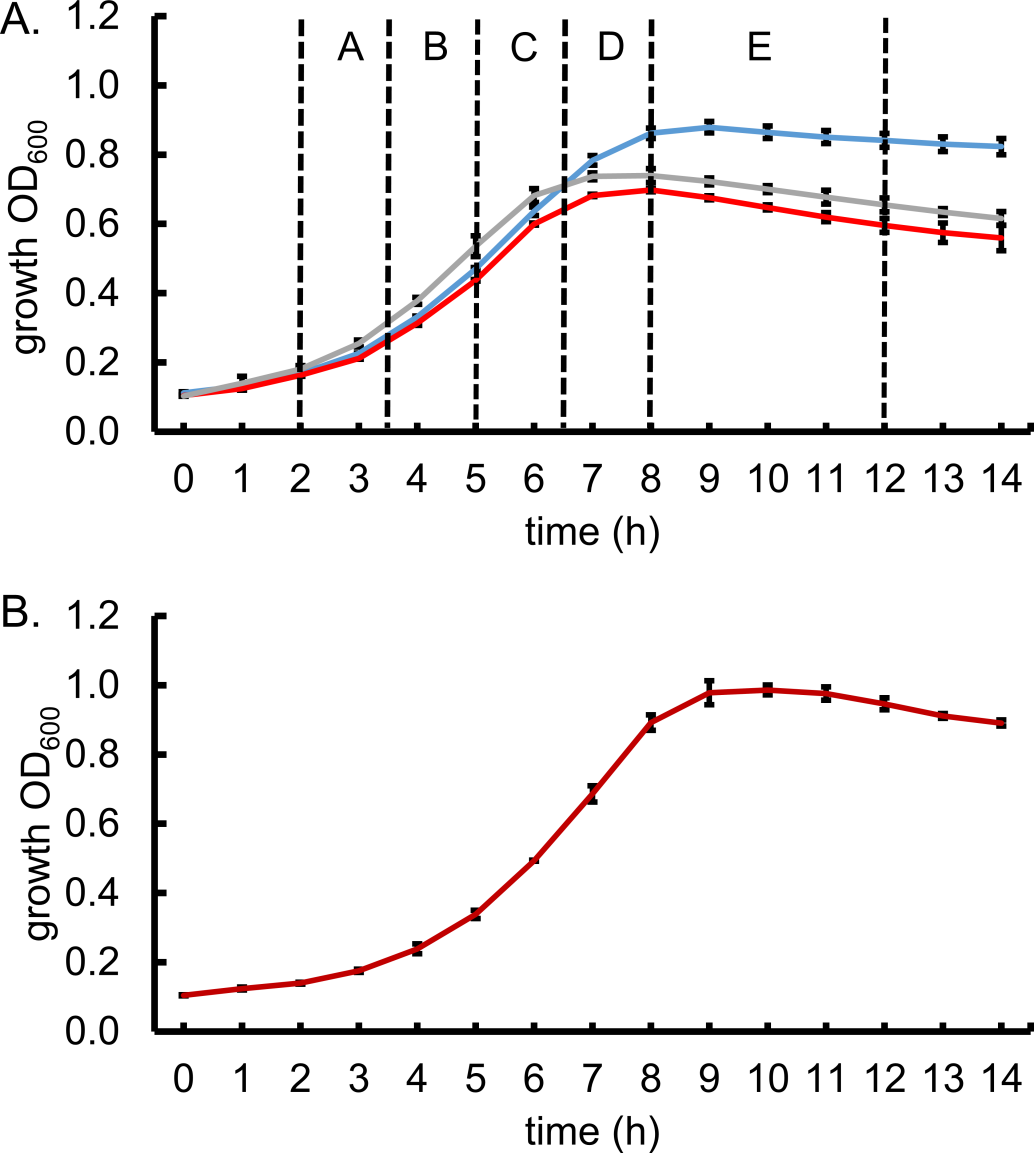
Reduced final OD_600_ attained during glucose fermentation by mutants lacking FHL-1. (**A**) Growth is represented with a linear *y*-axis to highlight the difference in final optical densities attained by the strains (see Fig. S1 for the corresponding semilogarithmic plots). The vertical dotted lines distinguish the lag phase (A); the early (B), mid- (C), and late (D) exponential growth phases; and the stationary phase (E), and these are referred to when describing the rest of the data. Strains include the parent, DH4100 (blue line), DH5000 (Δ*fhlA*) (light-red line), and HD700 (Δ*hycA-I*) (gray line). (**B**) Growth of DH5000 transformed with plasmid pSA32 (*fhlA*^+^). The growth experiments were performed with three biological and three technical replicates for each strain. The standard error of the mean is shown for each data point.

Anaerobic growth of the parental *E. coli* strain, DH4100, in defined M9-glucose minimal medium in a closed, batch-culture system showed a brief lag phase, followed by exponential growth for between 3 and 4 h (*μ* = 0.29; phases A, B, and C in Fig. 1A), before transitioning (phase D) into the stationary phase (phase E) after approximately 8 h (see also Fig. S1 for the corresponding semilogarithmic plot). The culture reached a final OD_600_ of nearly 0.9 (Fig. 1A). Growth of the *fhlA* mutant, DH5000, which lacks FHL-1 (14, 15), under the same conditions revealed similar lag and exponential growth phases to strain DH4100 (Fig. 1A); however, the transition into the stationary phase occurred earlier (6.5 h) and at a lower cell density (OD_600_ = 0.6). Introduction of plasmid pSA32 (*fhlA*^+^) (28) into strain DH5000 (Δ*fhlA*) restored fermentative growth (Fig. 1B; Fig. S1B) to a level comparable to that of the parental strain, DH4100. Moreover, the inability of strain DH5000 to generate H_2_ (29) could be restored by the introduction of plasmid pSA32 (*fhlA*^+^) (4.7 μmol H_2_ 0.2 mL^−1^ headspace, after 10 h of fermentative growth), which was a similar amount generated by DH4100 (4.8 μmol H_2_ 0.2 mL^−1^ headspace).

The lower final cell density attained by the *fhlA* mutant, DH5000, can be ascribed to the specific loss of the FHL-1 complex, as shown by the corresponding growth analysis of strain HD700 (Δ*hycA-I*), which lacks the genes encoding only the hydrogenase 3 structural subunits of FHL-1 (30). Growth of HD700 had a profile similar to that of strain DH5000 (Fig. 1A, gray line). Notably, both DH5000 and HD700 showed a steady reduction in cell density from about the 9 h time point. Introduction of plasmid pRBH (3), which includes a large, approximately 14 kbp DNA insert, including the complete *hycA-I* operon, into strain HD700 restored the ability of the strain to produce H_2_ (7.6 μmol H_2_ 0.2 mL^−1^ headspace, after 10 h of fermentative growth).

### Formate levels in the culture medium during fermentative growth

**Fig 2:**
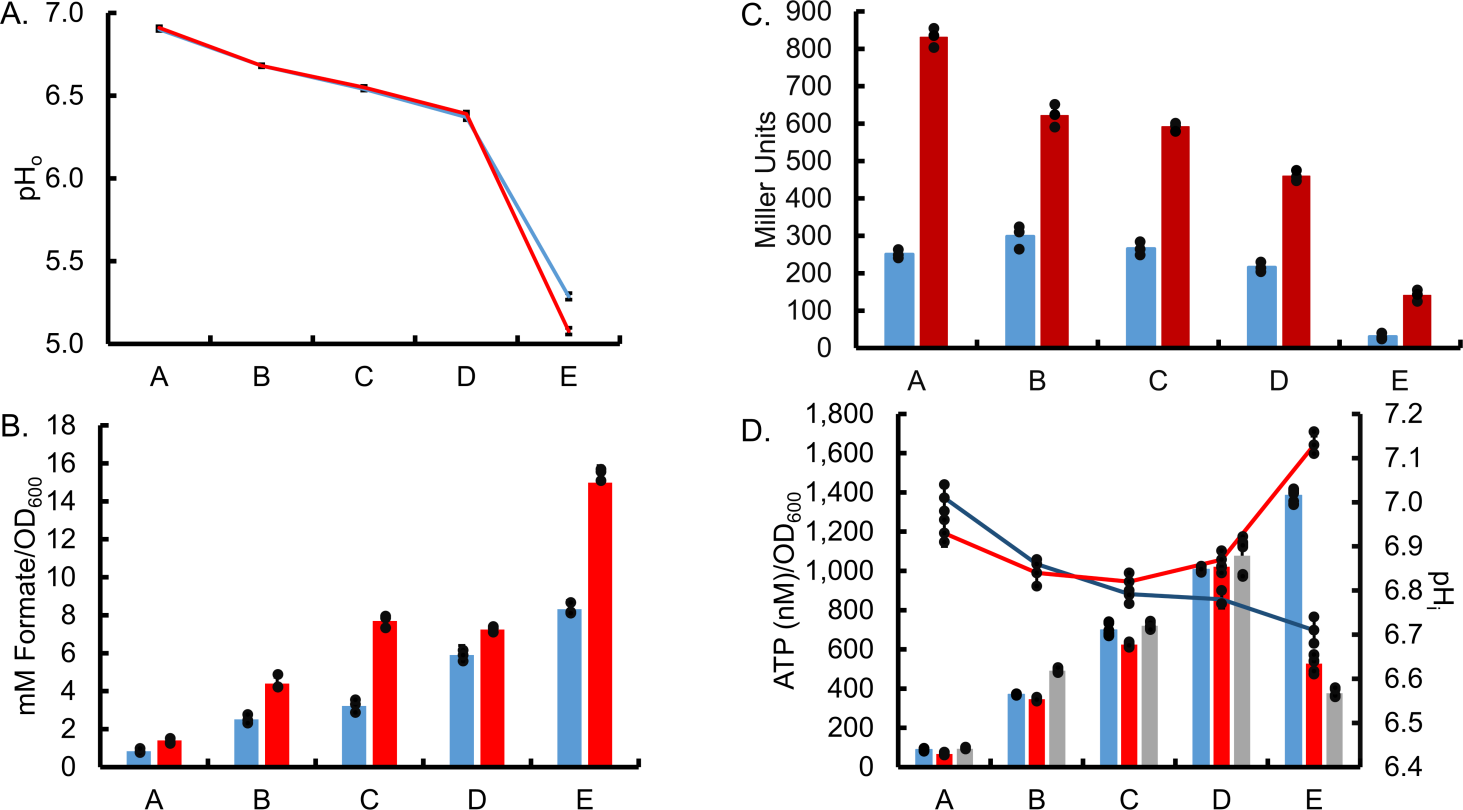
Differential ATP and pH parameters of DH4100 and DH5000 during glucose fermentation. Data are shown for pH_o_ (**A**) and formate concentration (**B**) in the culture medium during growth. Changes in intracellular formate levels, determined indirectly by measuring β-galactosidase enzyme activity (presented in Miller units), are shown in panel **C**, and pH_i_ and ATP concentration are shown in panel **D**. The letters A–E within the diagrams, which are indicated on the *x*-axes, correspond to the growth phases shown in Fig. 1, and the times at which samples were taken for determination of other cellular parameters are as follows: phase A, 3 h; phase B, 4 h; phase C, 6 h; phase D, 8 h; and phase E, 12 h. Data for strain DH4100 (parental) are shown as blue lines or histograms; those for DH5000 (Δ*fhlA*) are shown as light-red lines or histograms. The dark red histograms in panel C represent β-galactosidase enzyme activity measured in samples of the complemented strain DH5000/pSA32 (*fhlA*^+^); the gray histograms shown in panel **D** represent ATP levels in strain HD700 (Δ*hycA-I*). Data points are shown for three biological replicates. The standard error of the mean is shown for pH measurements shown in panel **A**, performed with three biological replicates.

The impaired fermentative growth of strain DH5000 (Δ*fhlA*) suggested initially that this might result from accumulated formate and H^+^ in the growth medium due to the lack of FHL-1 complex in the strain (29). Determination of the pH_o_ in the culture medium of both strains DH4100 and DH5000 revealed that their profiles were very similar, with the initial pH_o_ of 6.9 decreasing to 6.4 at the end of the exponential growth phase (phases A–D in Fig. 2A). However, upon entry into the stationary phase, the pH_o_ decreased sharply to 5.3 for the parental strain DH4100 (blue line) and 5.1 for DH5000 (light red line) (phase E in Fig. 2A). Introduction of plasmid pSA32 into the *fhlA* mutant DH5000 increased the pH_o_ in the culture medium to 5.5 in phase E of growth of the complemented mutant (data not shown), compared with a pH_o_ of 5.0 determined in the culture medium of the untransformed mutant.

Analysis of the extracellular formate concentrations during each phase of growth (A–D) showed that the culture medium of DH5000 had in it consistently higher levels of formate compared with the medium of DH4100 (Fig. 2B). After entry into the stationary phase (phase E), the culture medium of strain DH5000 had accumulated 15 mM formate OD_600_^−1^, excreted as formic acid (25, 31), compared with a value of 8 mM formate OD_600_^−1^ for the parental strain (Fig. 2B). The lower extracellular formate concentration in the culture of the parental strain is due to uptake by FocA and ultimately disproportionation by FHL-1. This result also suggests that the slightly lower pH in the culture medium of stationary-phase cells of DH5000 was due to enhanced accumulation of formic acid, mainly present as its dissociated species, relative to the parental strain.

### Sharp decrease in intracellular formate in stationary-phase cells

A recent study has shown that intracellular formate levels in the parental strain, DH4100, remain relatively constant during the growth of *E. coli* by glucose fermentation (32). Assessment of changes in intracellular formate levels in DH4100 cells (Fig. 2C), which were monitored indirectly by determining β-galactosidase enzyme activity using a chromosomally localized and formate-sensitive *fdhF_P_::lacZ* reporter fusion (17, 32), confirmed this finding. It was noted, however, that in stationary-phase cells of DH4100 (phase E), β-galactosidase enzyme activity decreased approximately 10-fold when compared with the activity in growing cells (Fig. 2C).

As strain DH5000 lacks the transcriptional regulator FhlA (29), this strain fails to induce β-galactosidase enzyme activity of the *fdhF_P_::lacZ* reporter fusion (data not shown). However, complementation of strain DH5000 with plasmid pSA32 restored to the mutant the ability to express the λ*fdhF*_P_::*lacZ* gene (Fig. 2C). Although the levels of β-galactosidase enzyme activity were approximately twofold higher than those of the parental strain, presumably due to the multicopy nature of the cloned *fhlA* gene on pSA32 (28), activity nevertheless also decreased to low levels in the stationary phase, suggesting intracellular formate consumption.

Together, the findings of this series of experiments indicate that formate accumulates to higher levels in the stationary-phase culture medium of strain DH5000 (Δ*fhlA*) (see Fig. 2B) because synthesis of FHL-1 cannot be induced, and thus formic acid cannot be disproportionated to H_2_ and CO_2_. In contrast, in stationary-phase cultures of the parental, FhlA^+^ strain, formate is reimported by FocA and disproportionated by FHL-1 to H_2_ and CO_2_ (20).

### Inverse correlation between intracellular pH and ATP levels in fermenting cells of the *fhlA* mutant

In order to determine what other physiological parameters might be altered in stationary-phase cells of DH5000, relative to DH4100, we next determined their intracellular pH (pH_i_) profiles during fermentation of glucose (Fig. 2D). At the initial stages of growth (phase A in Fig. 1), the pH_i_ in cells of the parental strain, DH4100, was 7 and that of the *fhlA* mutant was ~6.9. During growth phases B and C, the pH_i_ profiles for both strains were similar and decreased marginally to around 6.8 (Fig. 2D). However, upon entry into phase D, in which growth of DH5000 slowed significantly prior to entry into stationary phase, the pH_i_ rose slowly to approximately 6.9, while the pH_i_ of the parental strain, DH4100, decreased to slightly below 6.8. In stationary-phase cells (phase E in Fig. 2D), the pH_i_ in cells of the parental strain continued to decrease to 6.7, while that in cells of DH5000 increased to nearly 7.2. The pH_i_ values in stationary-phase cells of DH4100 and DH5000 were confirmed using the alternative pH-sensitive fluorescent probe, pHrodo Red (see Fig. S2).

These same cells were then used to determine the intracellular ATP levels (histograms in Fig. 2D). For comparative purposes, the intracellular concentration of ATP is presented relative to the optical density of the culture for the different growth phases. ATP levels for cells of both strains were similar in growth phases A–D, rising steadily from approximately 400 nM OD_600_^−1^ to 1 μM OD_600_^−1^ between growth phases B and D. However, in stationary-phase cells (phase E), the ATP concentration was nearly threefold lower in the *fhlA* mutant, DH5000, compared with the parental strain DH4100, which attained a level of approximately 1.4 μM ATP OD_600_^−1^ (Fig. 2D). Thus, these data indicate that the significant increase in pH_i_ in stationary-phase cells of the *fhlA* mutant, DH5000, correlated with a sharp decrease in intracellular ATP levels.

To determine whether this decrease in ATP level in stationary-phase cells of strain DH5000 was due specifically to loss of FHL-1 activity or whether it was caused by the loss of another FhlA-regulated function (14), the ATP levels throughout the growth phase were also determined in cells of the *hycA-I* mutant, HD700, which lacks only the FHL-1 complex (30). The data clearly show that the profile of ATP concentration in cells of strain HD700 during fermentative growth was similar to that of the *fhlA* mutant, DH5000, with the ATP concentration decreasing to approximately 400 nM OD_600_^−1^ in stationary-phase cells (Fig. 2D, gray histograms). Together, these data demonstrate that the specific loss of FHL-1 activity results in a dramatic decrease in ATP concentration, and this shows an inverse correlation with pH_i_ in stationary-phase cells of the *fhlA* mutant after growth by glucose fermentation.

### Complementation studies demonstrate recovery of parental levels of ATP

**Fig 3:**
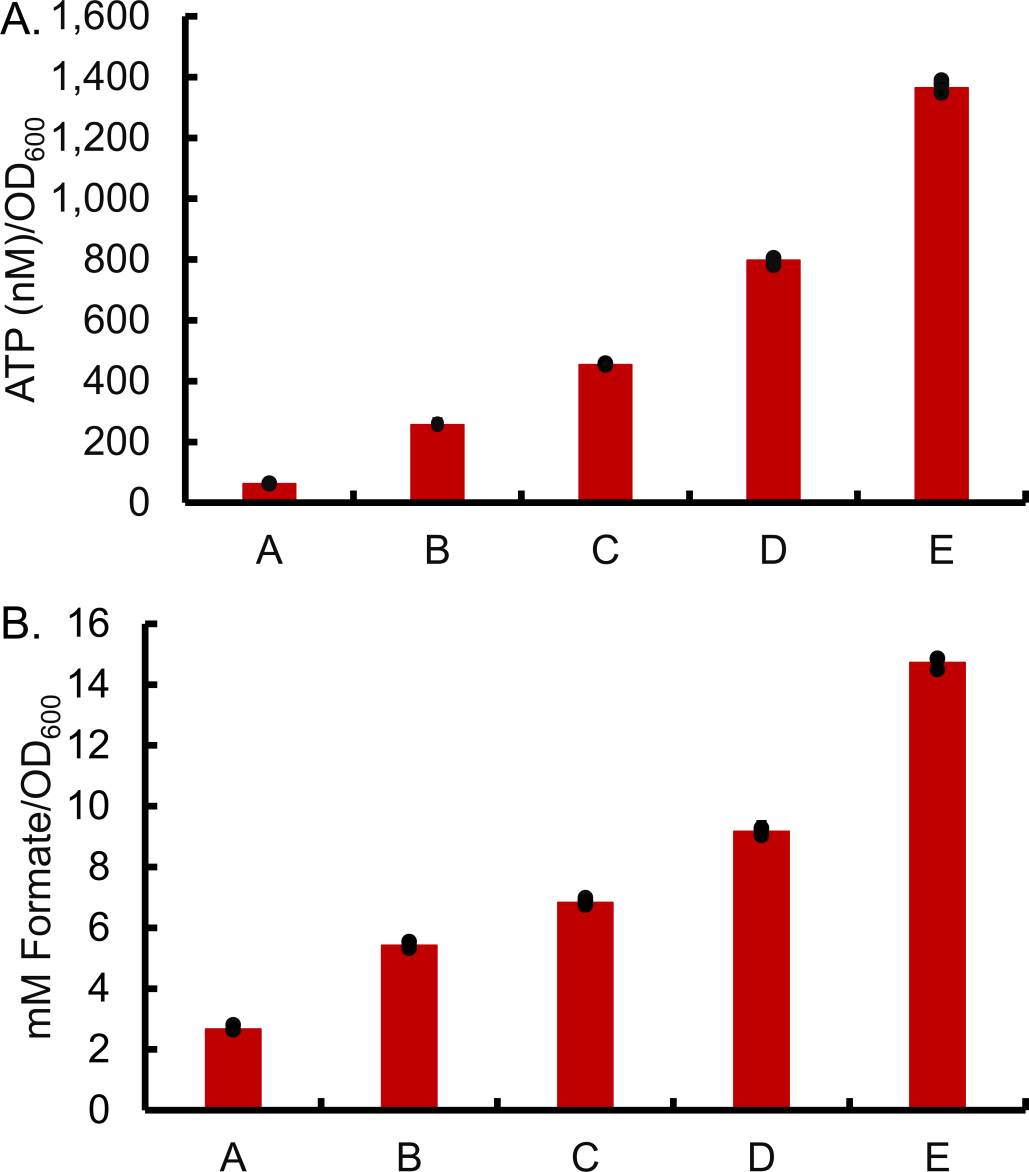
ATP and extracellular formate levels in strain DH5000 transformed with plasmid pSA32 (*fhlA*^+^). Data are shown for intracellular ATP levels (**A**) and for formate concentration (**B**) in the culture medium during growth of DH5000/pSA32. Parameters were determined at phases A, 3 h; B, 4 h; C, 6 h; D, 8 h; and E, 12 h. Data points are shown for three biological replicates.

To demonstrate that the decrease in ATP levels in stationary-phase cells of both DH5000 (Δ*fhlA*) and HD700 (Δ*hycA-I*) was specifically due to the lack of FHL-1 synthesis in each strain, intracellular ATP levels were determined throughout the growth phase for DH5000 (Δ*fhlA*) transformed with pSA32 (*fhlA*^+^) (Fig. 3A) and for HD700 (Δ*hycA-I*) transformed with pRBH (including the complete *hycA-I* operon) (Fig. S3A). The results show that ATP levels reached similar levels to those found in the parental strain, DH4100 (see also Fig. 2D), while the ATP level of 1.4 mM OD_600_^−1^ determined for DH5000/pSA32 in stationary-phase cells (Fig. 3A) was very similar to that measured for the parental strain, DH4100. In the case of HD700/pRBH, ATP levels were approximately 25% lower in the stationary phase (Fig. S3A, phase E) than those of the parental strain. Nevertheless, this amount of ATP was nearly threefold higher than determined for HD700 (Δ*hycA-I*) without plasmid pRBH (compare Fig. 2D).

The level of formate excreted from both complemented mutants, DH5000/pSA32 (Fig. 3B) and HD700/pRBH (Fig. S3B), was generally higher than that measured in the culture medium of the parental strain throughout growth, but in particular in the stationary phase (E) around 12 h. This is likely to be a reflection of the higher copy number of the genes reintroduced on the plasmids.

### Increased F_1_F_o_-ATPase activity in stationary-phase cells of strain DH5000

In an attempt to determine the cause of the decrease in cellular ATP levels in stationary-phase cells of the *fhlA* mutant, both the total and *N*,*N*′-dicyclohexylcarbodiimide (DCCD)-sensitive ATPase activities in membrane vesicles derived from cells of the parental strain, DH4100, and the *fhlA* mutant, DH5000, were determined. DCCD inhibits the activity of the F_1_F_o_-ATPase enzyme (33). The results shown in Table 1 reveal that in membrane vesicles of late-exponential phase (phase C) cells from the *fhlA* mutant, the total ATPase activity was 143 ± 15 nmol Pi min^−1^ μg^−1^ of protein, which was approximately 80% of the activity that was determined for vesicles from the parental strain (176 ± 25 nmol inorganic phosphate [Pi] min^−1^ μg^−1^ of protein) in the same growth phase. After DCCD treatment, the activity attributable to F_1_F_o_-ATPase was 134 and 97 nmol Pi min^−1^ μg^−1^ for the parental strain and the *fhlA* mutant, representing a contribution of 76% and 68% of the total activity for each strain, respectively (Table 1). In membrane vesicles derived from stationary-phase cells (phase E), the activity attributable to F_1_F_o_-ATPase was calculated to be 74 and 112 nmol Pi min^−1^ μg^−1^ for the parental strain and the *fhlA* mutant, representing a contribution of 55% and 77% of the total activity, respectively. This difference in percentage contribution indicates that the F_1_F_o_-ATPase activity was approximately 40% higher in strain DH5000, relative to the activity in the parental strain.

**TABLE 1:** ATPase activity in membrane vesicles

Strain and growth phase^*a*^	Total ATPase activity (nmol Pi min^−1^ μg^−1^)	Total ATPase activity + DCCD (nmol Pi min^−1^ μg^−1^)	Activity due to F_1_F_o_-ATPase (nmol Pi min^−^¹ μg^−^¹)
DH4100 (phase C)	176 ± 25	42 ± 15	134
DH4100 (phase E)	135 ± 35	56 ± 5	74
DH5000 (phase C)	143 ± 15.1	46 ± 14.1	97
DH5000 (phase E)	145 ± 15.3	33 ± 11	112

^*a*^ Growth phases C and E represent late-exponential phase and stationary phase, respectively (see Fig. 1).

### Effect of dissipation of the proton gradient on ATP levels

**Fig 4:**
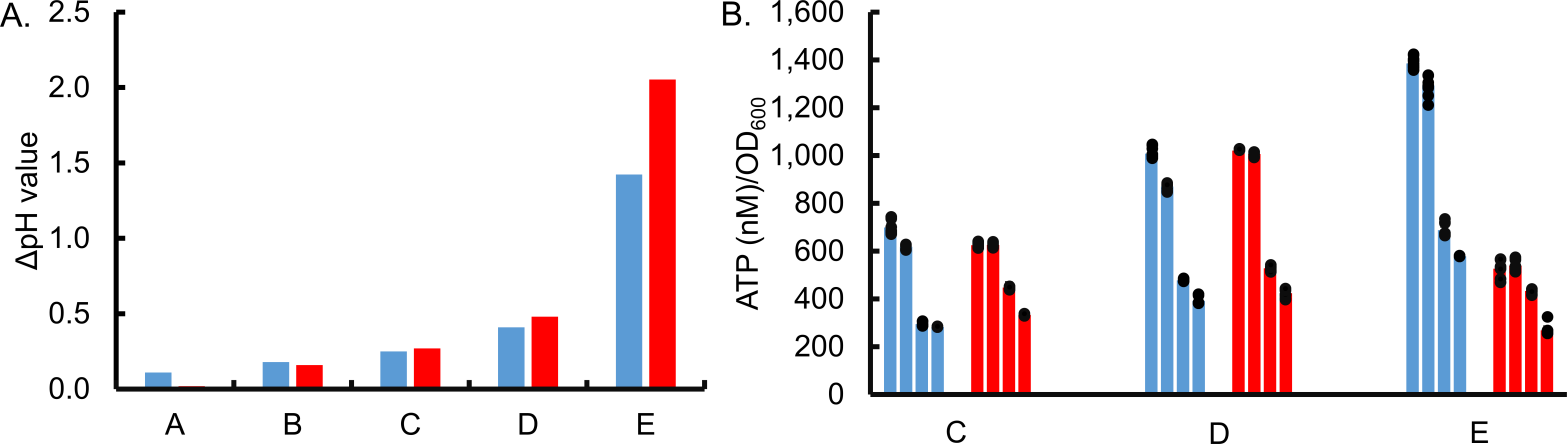
Comparison of external and internal pH values and effect of CCCP on ATP levels during glucose fermentation. (**A**) The ΔpH (pH_i_–pH_o_) during the different phases of growth. In panel **B**, the effect of transient dissipation of the proton gradient on ATP levels in the cells during different phases of growth is shown. Each set of four histograms represents (from left to right) no addition, addition of 5 μM, addition of 50 μM, or addition of 100 μM CCCP on ATP concentration. The letters A–E within the diagrams, which are indicated on the *x*-axes, correspond to the growth phases shown in Fig. 1 and correspond to A, 3 h; B, 4 h; C, 6 h; D, 8 h; E, 12 h. While data for strain DH4100 (parental) are shown as blue histograms, those for DH5000 (Δ*fhlA*) are shown as red histograms. Data points are shown for three biological replicates.

Comparing ΔpH (pH_i_ minus pH_o_) throughout the growth phase for both strains (Fig. 4A) revealed that, while during growth (phases A through D) both strains exhibited a similar, comparatively low ΔpH of approximately 0.1 to 0.2 units in phases B and C, this increased to 0.5 units in phase D at the beginning of the transition to stationary phase. In the stationary phase, the ΔpH was considerably higher, reaching nearly 1.5 pH units for the parental strain (DH4100), and 2.2 pH units for the *fhlA* mutant, DH5000 (Fig. 4A). These data suggest that the ΔpH formed a major component of the proton motive force in stationary-phase cells of fermenting cultures.

To determine the impact of dissipating the proton gradient on intracellular ATP levels, aliquots of cells from both strains in growth phases C, D, and E (see Fig. 1) were incubated for 4 min with different concentrations of the uncoupler carbonylcyanide-*m*-chlorophenyl hydrazone (CCCP) (see MATERIALS AND METHODS before determining the intracellular ATP levels of the cells (Fig. 4B). Data for cells of both strains in growth phases C and D showed very similar effects of CCCP, whereby in phase D, an approximate 50% reduction in ATP levels to 400 nM OD^−1^ was observed. In stationary-phase cells (Fig. 4B, growth phase E), a difference in response between the cells from the two strains was apparent. Incubation of cells of the parental strain, DH4100, with 50 μM CCCP reduced ATP levels to approximately 600 nM OD^−1^, close to the level of ATP in untreated cells of the *fhlA* mutant, DH5000 (Fig. 4B). This result indicates that dissipation of the proton gradient in stationary-phase cells of the parental strain, despite the presence of an active FHL-1 complex, caused increased hydrolysis of intracellular ATP to a level close to that observed for the FHL-1-negative mutant, DH5000, without the addition of an uncoupler (Fig. 4B).

### Bicarbonate restores growth, ATP levels, and pH_i_ of the *fhlA* mutant to near-parental levels

**Fig 5:**
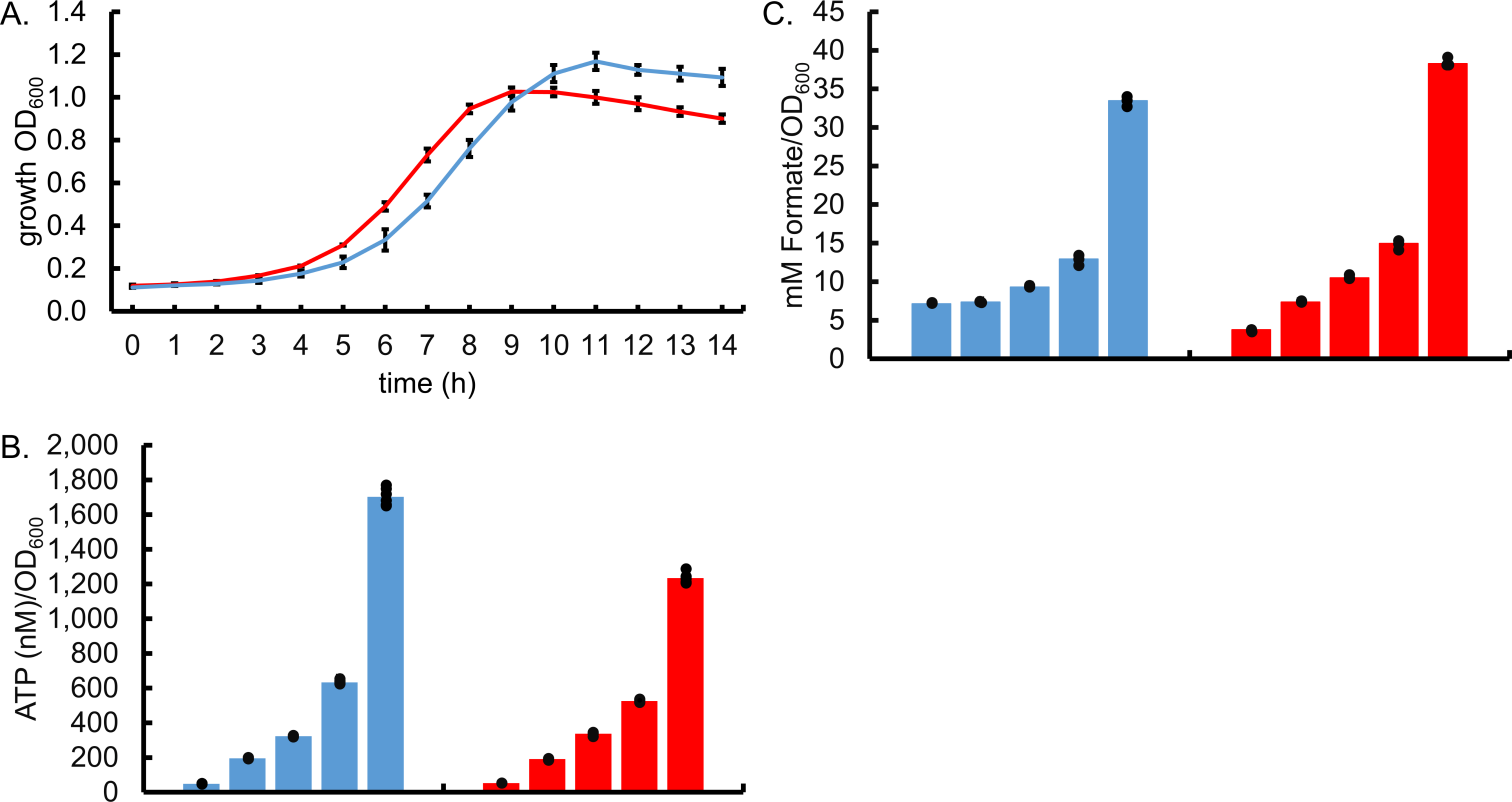
Fermentative growth in the presence of 50 mM sodium bicarbonate restores ATP levels and increases formate export. (**A**) Anaerobic growth of DH4100 (blue line) and DH5000 (light-red line) in M9-glucose supplemented with 50 mM sodium bicarbonate. ATP levels (**B**) and extracellular formate production (**C**) were determined in phases A, 3 h; B, 4 h; C, 6 h; D, 8 h; and E, 10 h. Blue histograms show data for DH4100; light-red histograms show data for DH5000 (Δ*fhlA*). The growth experiment was performed with three biological and three technical replicates for each strain. The standard error of the mean is shown for each data point. Data points in panels B and C are shown for three biological replicates.

Due to the catalytic centers of the FHL-1 complex being located in the cytoplasm, the inability of a mutant, such as DH5000 (Δ*fhlA*), to synthesize this complex means that these cells are no longer capable of generating intracellular CO_2_. Attempts to compensate for this loss of CO_2_ production by culturing DH4100 and DH5000 under an atmosphere of 80% N_2_/20% CO_2_ failed to restore significantly improved anaerobic growth to the *fhlA* mutant compared to the parental strain DH4100 (data not shown). However, anaerobic growth of the parental strain, DH4100, and its *fhlA* mutant, DH5000, in M9-glucose medium supplemented with 50 mM sodium bicarbonate significantly increased the cell density attained by cultures of both strains (Fig. 5A). The culture of DH4100 attained a maximal OD_600_ of 1.15 after 11 h of growth, while the culture of DH5000 attained a maximal OD_600 nm_ of 1.1 after 9 h. This represents an increase of approximately 40% in the cell density of the *fhlA* mutant compared to that without added bicarbonate (compare Fig. 1).

Determination of ATP levels throughout the growth phase for both strains cultivated in the presence of 50 mM bicarbonate revealed parallel increases in ATP during growth phases A–D for both strains (Fig. 5B), while the level of ATP in the stationary phase (phase E) for DH4100 was determined to be 1.7 mM OD_600_^−1^ and that for DH5000 was 1.25 mM OD_600_^−1^. This represents a 2.5-fold increase in intracellular ATP levels for strain DH5000 compared with the level in cells grown without bicarbonate supplementation.

After growth to stationary phase (phase E, 12 h) in the presence of bicarbonate, the pHi in cells of DH5000 was determined to be 6.73, while that measured in cells of DH4100 after 12 h of growth was 6.81. Taken together, these findings indicate that high levels of exogenously supplemented bicarbonate cause an increase in ATP levels and a concomitant decrease in pH_i_, strongly suggesting that increasing intracellular CO_2_/bicarbonate levels minimizes the requirement for a FHL-1 complex during mixed-acid fermentation.

To exclude the possibility that the restoration of growth and near-wild-type phenotypes of other cellular parameters in the *fhlA* mutant by bicarbonate supplementation to the growth medium was a consequence of increased pH_o_, the pH_o_ was determined during cultivation of both strains throughout the growth phase (data not shown). Comparison of pH_o_ during growth in the presence and absence of 50 mM bicarbonate revealed for both strains a similar and parallel, approximately 0.4–0.5 pH unit increase during the exponential-phase growth, increasing to ΔpH_o_ of 0.8 units in the stationary phase.

As a further control, growth of both strains was analyzed at pH 8 in M9-glucose medium (Fig. S4). The increase in initial pH of the growth medium did not restore a parental growth phenotype to DH5000 (Δ*fhlA*) when compared with growth of DH4100 under the same conditions, clearly indicating that the improved growth of strain DH5000 in the presence of 50 mM bicarbonate was not due to the marginal pH_o_ increase caused by bicarbonate supplementation.

### Increased formate efflux during fermentative growth in the presence of high bicarbonate

If bicarbonate can substitute as a source for formate-derived intracellular CO_2_, then it would be expected that extracellular formate levels should concomitantly increase in cultures of strains grown under fermentative conditions in the presence of 50 mM bicarbonate. To test this, we measured extracellular formate levels for both strain DH4100 and its isogenic *fhlA* mutant, DH5000. The levels of formate in the culture medium were comparable for both strains (Fig. 5C). However, compared with cultivation in the absence of bicarbonate (compare with Fig. 2B), extracellular formate levels were increased by nearly fivefold (34 mM OD_600_^−1^) in stationary-phase cultures after growth of DH4100 in the presence of bicarbonate, while the concentration of formate was more than doubled (38 mM OD_600_^−1^) in stationary-phase cultures of DH5000. These results confirm that high concentrations of exogenously supplied bicarbonate lead to reduced formate consumption by the fermenting cells.

## DISCUSSION

Due to pyruvate being cleaved homolytically to acetyl-CoA and formate by PflB during mixed-acid fermentation, enterobacteria like *E. coli* fail to generate CO_2_ using a classic decarboxylation reaction. Instead, they rely on the FHL-1 complex to disproportionate formic acid into H_2_ and CO_2_. We show in this current study that *E. coli* mutants unable to synthesize an active FHL-1 complex not only fail to generate CO_2_ but also have problems maintaining intracellular pH homeostasis and ATP levels in stationary-phase cells. Based on the findings of earlier studies (6, 25, 34), it had been implicated, but not clearly demonstrated, that FHL-1 has a role in maintaining pH homeostasis. In the absence of a functional FHL-1 complex, influx of formic acid via FocA (34) and, to a certain extent, through diffusion at low pH_o_ (6) can occur. We could also show that in order to offset any resulting acidification of the cytoplasm due to dissociation of protons, enhanced ATP hydrolysis by the F_1_F_o_-ATPase increased proton translocation across the cytoplasmic membrane, which resulted in alkalinization of the cytoplasm. This overcompensation through activation of the F_1_F_o_-ATPase resulted in substantial ATP hydrolysis in cells of the *fhlA* mutant, compared with cells of the parental strain (Fig. 4). Excessive ATP hydrolysis has the consequence that the final cell density attained by the *fhlA* mutant is reduced. The fact, however, that addition of high concentrations of bicarbonate to the growth medium essentially complemented, in large part, the FHL-1-negative phenotype indicates that it is the lack of intracellularly generated CO_2_ that is the direct cause of both the alkalinization of the cytoplasmic pH and the reduction in ATP levels in the mutant. Bicarbonate, which derives from the hydration of CO_2_ to carbonic acid either by carbonic anhydrase or via rapid equilibration at pH values above 7 (23), is essential for carboxylation reactions in stationary-phase cells, particularly the acetyl-CoA carboxylase reaction, which is necessary to stabilize membrane lipids in these cells (23). Based on our findings, an insufficient level of CO_2_/bicarbonate appears to be linked to activation of ATP-hydrolyzing F_1_F_o_-ATPase activity, currently by an unknown mechanism, which results in an increased intracellular pH, ultimately shifting the equilibrium towards bicarbonate anion formation. Moreover, carbonic anhydrase activity is also increased in stationary-phase cells and by alkaline pH (23). Of course, high exogenous bicarbonate concentrations present an artificial situation, and the role of maintaining optimal intracellular CO_2_/bicarbonate, as well as ultimately ensuring pH homeostasis and optimal ATP levels, is adopted under physiological conditions by FHL-1.

**Fig 6:**
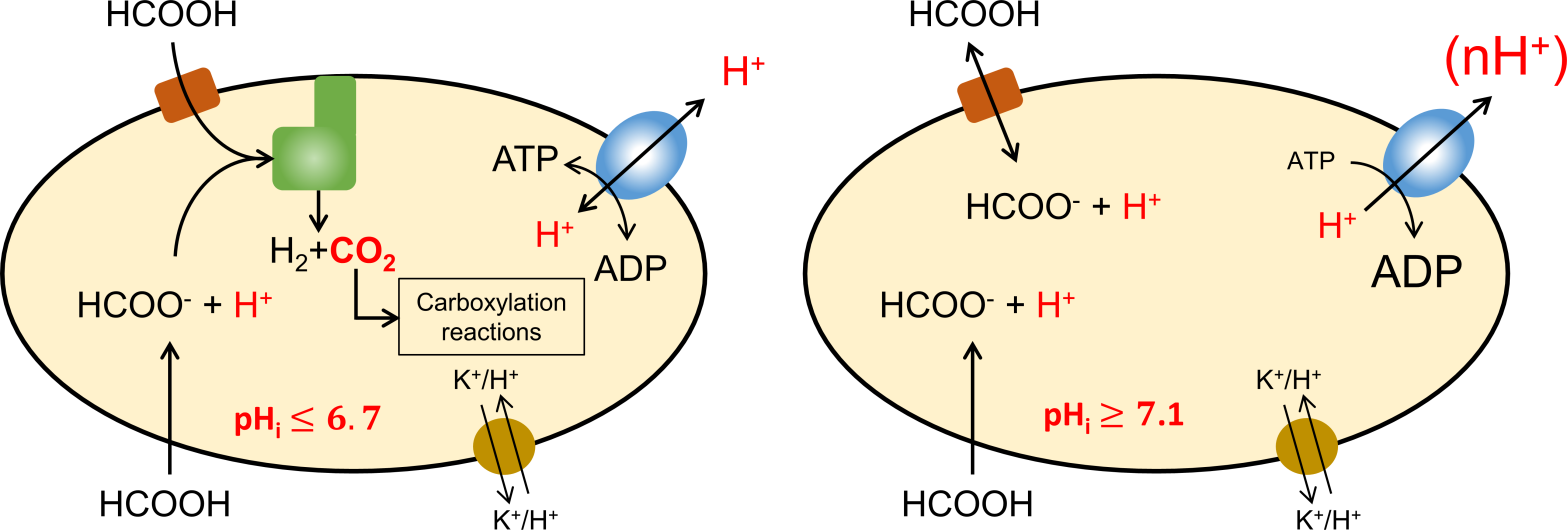
Schematic representation of the consequences of the presence (left) and absence (right) of FHL-1 on cellular ATP and CO_2_ levels and on intracellular pH_i_. The scheme shows a highly stylized and simplified *E. coli* “wild-type” cell, including FHL-1 (green) on the left. The arrows indicate the direction of formic acid or ion movement. Increased ATP hydrolysis by the F_1_F_o_-ATPase (blue oval) is indicated by enhanced ATP hydrolysis to ADP in the “cell” lacking FHL-1 on the right. The red rectangle represents FocA, and the brown sphere represents potassium-proton exchangers.

Induction of FHL-1 synthesis in the parental strain is induced in response to the presence of formate (14), resulting in both the internally generated formate and any imported exogenous formic acid (25) swiftly being converted to the neutral gases H_2_ and CO_2_. While H_₂_ escapes to the periplasm or the immediate environment surrounding the cell, the provision of intracellular CO_2_, when trapped as bicarbonate, with concomitant maintenance of both pH homeostasis and ATP levels, facilitates improved growth and stationary-phase survival (25, 26). Consequently, FHL-1 contributes indirectly to energy metabolism in the stationary-phase cell by hindering ATP hydrolysis via F_1_F_o_-ATPase, with the concomitant maintenance of cytoplasmic pH at 6.7–6.8 (see Fig. 6).

It is well established that the pH_i_ of respiring *E. coli* cells is maintained within the range of 7.4–7.6 (35, 36), which is considerably higher than the pH_i_ of fermenting cells. A pH_i_ of 7.5 is also ideal for maintaining a high ratio of the membrane-impermeant bicarbonate anion relative to freely diffusible CO_2_ (23). Thus, when the results of our study clearly showed that during growth by glucose fermentation the pH_i_ is maintained close to 6.8, this was initially somewhat unexpected. However, these results substantiate similar recent findings for cells cultured by glucose fermentation at an initial pH_o_ of 7.5 and of 5.5, which also determined the pH_i_ during growth under these conditions to be in the range of 6.8; notably, in those studies, an acridine orange-based method was used to monitor pH_i_ (37, 38). The lower pH_i_ during mixed-acid fermentation of *E. coli* can be accounted for by high-level formate generation via PflB (16) and its comparatively high intracellular concentration (14, 15). Moreover, this also explains the high apparent *K*_d_ for formate of FhlA (5 mM) (39) and the high *K*_m_ for formate (26 mM) of the Fdh-H subunit of the FHL-1 complex (40). In contrast, homolactate fermenters, such as *Lactococcus cremoris* and *Lactococcus lactis* (formerly named *Streptococcus*), have a pH_i_ of roughly 7.5 and achieve this by maintaining a low cytoplasmic lactate (p*K*_a_ = 3.86) concentration through rapid excretion of each lactate in symport with two protons, which results in maintenance of the higher pH_i_ (41).

Of the organic acids produced during mixed-acid fermentation by *E. coli* with glucose as a carbon source, only formic acid is reimported, essentially almost completely. This is feasible only because of the strict coordination of gene expression, enzyme activity, and physiology between PflB, FHL-1, and the formate channel/transporter FocA in direct response to intracellular formate levels (14, 20, 34). FocA maintains a relatively constant intracellular formate level (32). It achieves this by controlling the efflux or import of the acid in response to formate production by PflB, its relative rate of consumption by FHL-1, and presumably by the increased bicarbonate requirement for carboxylation reactions, particularly in the stationary phase (23). Disturbance of this finely balanced system, e.g., by preventing FHL-1 activity, results in a lack of bicarbonate and a breakdown in cellular pH and ATP homeostasis (14, 34). This system also represents a lucid example of an organic acid degradation mechanism previously proposed to serve as a means of survival by heterotrophic acidophiles (42).

The signal to which the ATP-hydrolyzing activity of F_1_F_o_-ATPase in fermenting cells responds still remains somewhat of an enigma, and despite the fact that the ATPase itself has been suggested to sense pH changes (36, 43), the findings of the current study point to CO_2_ or bicarbonate levels as possibly being the signal that is sensed. The fact that acetyl-CoA carboxylase functions optimally in the range of pH 7.5 (24, 44, 45) suggests that it might also somehow be involved in sensing bicarbonate levels. How this signal might be transduced mechanistically to control F_1_F_o_-ATPase activity will, however, require further experimental study.

Formic acid uptake may also be involved, albeit indirectly, in transmitting a signal, as current data suggest that formate plus a proton are imported into stationary-phase cells via FocA (25, 46). This will lead to a potential charge-imbalance in the membrane potential if formate cannot be metabolized but protons are expelled, which might in turn require charge compensation by the uptake of K^+^ ions. Findings of initial studies (47) have provided evidence in support of this. While the different Trk transporter systems, acting both as K^+^/H^+^ exchangers and symporters, can help depolarize the membrane potential, these systems nevertheless likely do not have a major bearing on pH homeostasis (36) in the same manner that the F_1_F_o_-ATPase has during fermentation.

The demonstration that dissipation of the proton gradient by CCCP, with the concomitant rapid reduction in ATP levels in the parental strain (despite FHL’s presence) to levels similar to those determined for the *fhlA* mutant without CCCP treatment (Fig. 4B), strengthens the suggested coupling between FHL-1 and F_1_F_o_-ATPase activities (48) with formic acid uptake by FocA. Indeed, Pinske and Sargent (9) also demonstrated an almost 60% reduction in FHL-1-dependent H_2_ production upon treatment of *E. coli* with CCCP, further substantiating that FHL-1 activity, and thus CO_2_/bicarbonate production, is reliant on the proton gradient and is coupled with energy metabolism. Moreover, this proposed coupling between FHL-1 and F_1_F_o_-ATPase is also supported by the earlier observation that mutants of *Salmonella enterica* serovar Typhimurium (formerly *S. typhimurium*) and *E. coli* lacking a functional F_1_F_o_-ATPase failed to produce H_2_ by the FHL-1 complex (27). Whether the proton gradient drives formate uptake by FocA for delivery to FHL-1 mainly to enhance CO_2_/bicarbonate production should also be considered. It is perhaps notable that there is currently no description of a bicarbonate transport system in *E. coli*. In earlier experiments, we noted that 10 mM bicarbonate was insufficient to complement the phenotype of the *fhlA* mutant (C. Erdmann and R. G. Sawers, unpublished data), so the fact that such high bicarbonate concentrations are required to effect complementation suggests either that bicarbonate enters the cells via a low-affinity and unspecific transport system or that the high extracellular bicarbonate concentration creates a high local and diffusible CO_2_ concentration, allowing its entry into the cytoplasm and then its entrapment as bicarbonate via carbonic anhydrase (23).

The requirement for FHL-1 to be membrane associated to produce H_2_ *in vivo* (6, 9) has led to speculation that the HycCD (7, 8, 30) anchor subunits of FHL-1 facilitate proton translocation and thus contribute directly to energy conservation. To date, an energy-conserving function for FHL-1 lacks experimental substantiation, and indeed, studies by Pinske and Sargent (9) have provided strong evidence that this function cannot be attributed to FHL-1. Instead, the findings of the current study suggest that membrane attachment is a consequence of coupling between FocA, which delivers the substrate for FHL-1, and the F_1_F_o_-ATPase, which balances intracellular pH and optimizes ATP levels, presumably in dependence on the cellular requirement for CO_2_/bicarbonate (Fig. 6). Together, this suggests that the FHL-1 class of enzymes, with ‘stripped-down’ dual-anchoring HycCD subunits, mainly function to deliver CO_2_ and thus, indirectly, help maintain pH homeostasis and, as a consequence, indirectly maintain ATP levels, while the FHL-2 class with its full complement of subunits in the membrane domain may well have an energy-conserving function similar to complex I (6, 10–12). The functional association of FocA with FHL-1, and indirectly with the F_1_F_o_-ATPase, helps ensure formate (32) and ultimately bicarbonate homeostases. It has been known for a century that H_2_ production by *E. coli* is formate-dependent and that this occurs at a pH_o_ below 6.5 (2, 6, 14). The findings of this study highlight FHL-1′s likely principal role in the previously overlooked generation of CO_2_/bicarbonate for carboxylation reactions.

## MATERIALS AND METHODS

### Strains, plasmids, and growth conditions

The strains of *E. coli* used in this study include DH4100 (49), which is a derivative of MC4100 (F^−^ *araD* Δ(*argF lac*) *U 169 ptsF25 deoC1 relA1 fblB530 rpsL 150 λ*^−^; 50) and has a single-copy *λ*(*fdhF_P_::lacZ*) fusion. DH5000 is like DH4100, but has a deletion in the *fhlA* gene (29). HD700 is derived from MC4100, but has a deletion in the complete *hyc* operon (30). The plasmids used included pSA32 (*fhlA*^+^) (28) and pRBH (*hycA-I*^+^) (3).

All strains were grown anaerobically in liquid M9-minimal medium, pH 7.0, with 0.8% (wt/vol) glucose as a carbon source, and at 37°C, exactly as described (51). Where indicated, the medium was supplemented with 50 mM sodium bicarbonate (Carl Roth, Karlsruhe, Germany). Anaerobic growth analysis was carried out in 96-well microtiter plates, as previously described (51). Kanamycin was supplemented to the growth medium at a final concentration of 50 μg mL^−1^ for selection of strains DH4100 and DH5000.

Cultivation of the strains for all other experiments was done in the same M9-glucose minimal medium either in 15 mL Hungate tubes filled with 5 mL of medium, or in crimped serum bottles (250 mL) filled with 50 mL of medium, under a N_2_ atmosphere (51).

### Determination of intracellular ATP levels

Intracellular ATP levels were determined using the luciferase-based ATP Bioluminescence Assay Kit CLS II (Roche Diagnostics, Basel, Switzerland), and whole cells were sampled at different stages of growth. The ATP standard delivered in the kit was suspended in double-distilled H_2_O exactly as described in the manufacturer’s instructions and was used to prepare a standard curve, which was linear in the range between 5 and 250 nM. The luciferase enzyme solution was prepared and transferred to black 1.5 mL reaction tubes (Rotilabo Carl Roth GmbH, Germany) and maintained on ice. This solution was used within 24 h of preparation, during which time it remained stable. Aliquots (200 μL) of TE buffer (100 mM Tris, 4 mM EDTA, pH 7.75) were added to 1.5 mL reaction tubes, which were placed in a thermoblock (Thermo Shaker TS1; Biometra, Germany) and equilibrated for 7 min at 98°C. After equilibration, 50 μL of cell suspension was added directly to a reaction tube, and incubation was continued for a further 7 min to ensure complete cell lysis. Lysed cells and precipitated cell material were removed by centrifugation for 3 min at 13,000 rpm in a benchtop centrifuge. A 200 μL aliquot of the supernatant fraction was placed in a fresh 1.5 mL reaction tube, and the centrifugation step was repeated for a further 2 min at 13,000 rpm. Then, a 155 μL aliquot of the supernatant from this second centrifugation step was transferred to a fresh reaction tube, and the sample was maintained in the dark at 4°C until the assay. Immediately prior to assay, the tubes were equilibrated at room temperature for 15 min in the dark, and then 50 μL was added to a well of a white-walled 96-well microtiter plate (Greiner Bio-One International GmbH, Austria). The use of a white-walled 96-well microtiter plate significantly reduced autofluorescence of the samples. The 96-well microtiter plate was placed in a plate reader (Tecan Spark; Tecan Trading AG, Switzerland), and the temperature of the samples was equilibrated by incubating at 24°C for 5 min. The reaction was started by the addition of 50 μL of freshly prepared and equilibrated luciferase solution. Prior to recording the emission of light, the plate was shaken in the plate reader at 180 rpm for 20 s to ensure complete mixing of the samples. For each sample, five measurements were taken between 533 and 593 nm to ensure reproducibility, and the peak measurement at 562 nm was used for determination of the ATP concentration in the sample. Experiments were performed with three biological replicates, and each biological replicate was determined with three technical replicates. Data are presented as the standard deviation of the mean.

To test the effect of the uncoupler CCCP on ATP levels, strains were cultivated anaerobically in M9-glucose medium in 15 mL Hungate tubes to the indicated phase of growth. CCCP dissolved in DMSO was added to final concentrations of 5, 50, and 100 µM, and incubation was continued for 4 min, as described (52), after which cells were immediately placed on ice and ATP levels determined.

### Analysis of changes in intra- and extracellular formate levels

Changes in intracellular formate levels were determined indirectly by measuring the β-galactosidase enzyme activity exactly as previously described (17, 51). Determination of formate concentration in the growth medium was done by high-performance liquid chromatography and was monitored at 210 nm, exactly as described (17). The formate concentration was calculated with reference to the optical density (OD_600 nm_). The experiments were performed with three biological replicates, each with three technical replicates and data are presented as standard deviation of the mean.

### Measurement of extracellular and intracellular pH

Extracellular pH_o_ was measured in the culture medium using a 766 Calimatic pH meter (Knick Elektronische Messgeräte GmbH & Co. KG, Germany) equipped with a WTW pH SenTix Mic microelectrode (Fisher Scientific GmbH, Germany).

Intracellular pHi was determined using 2′,7′-bis(2-carboxyethyl)-5-(and 6)-carboxyfluorescein-acetoxymethyl-ester (BCECF-AM; Thermo Fisher Scientific, USA). Due to the lipophilic group of BCECF-AM, it does not fluoresce, and with a pKa of 6.98, it is membrane permeant at physiological pH. Non-specific esterases in the cytoplasm cleave the lipophilic blocking group, releasing charged BCECF, which shows green fluorescence (53). Cell suspensions (1 mL) were sampled at the indicated time points during growth and placed in a 1.5 mL reaction tube containing a dilution of a BCECF-AM solution calculated to be sufficient for the amount of cells added (0.75 μM BCECF-AM for OD_600_ = 0.3; 1.5 μM BCECF-AM for OD_600_ = 0.6; 3 μM BCECF-AM was used for all OD_600_ higher than 0.6), and the tubes were mixed vigorously and incubated for 1 h at 37°C in the dark. Subsequent to incubation, the samples in the tubes were centrifuged for 10 min at 9,000 rpm at 4°C. The supernatant was decanted and discarded, and the cell pellet was resuspended and washed in 50 mM Tes-Mes-Bis-Tris (TMBT) buffer (each at 50 mM), pH 7.0. For cell samples with an OD_600_ higher than 0.6, this washing step was repeated three times to ensure that any residual and non-specifically adsorbed BCECF-AM was removed from the cells.

After the last washing step, cell pellets were suspended in 300 μL of 50 mM TMBT buffer, pH 7.0, for further analysis. To prepare the calibration curve, cell samples were suspended and washed in buffer solutions covering the pH range of 5–7.5, finally being resuspended in 240 μL of 50 mM TMBT, buffered at the appropriate pH. KCl-CCCP stock solution (60 μL) (750 mM KCl and 50 μM CCCP) was added to this suspension to allow the internal pH to equilibrate with the pH of the TMBT buffer used to generate the calibration curve (54). These samples were then incubated in the dark for 30 min at 37°C. After this period of incubation, a 150 µL aliquot of each sample was placed in a well of a black-walled 96-well microtiter plate. The plate was then placed in a Tecan Spark plate reader, and samples were measured using excitation wavelengths of 440 and 490 nm and an emission wavelength of 505 nm over a period of 30 min at 37°C. Each sample was determined using three biological replicates, with each replicate performed in technical duplicate. Data are presented as the standard deviation of the mean.

### Measurement of ATPase enzyme activity

Total ATPase enzyme activity was determined in membrane vesicles, exactly as described (31). Briefly, right-side-out membrane vesicles were made from freshly prepared spheroplasts. Total ATPase activity was determined by measuring the amount of Pi released by incubating aliquots of membrane vesicles in 50 mM Tris–HCl, pH 7, containing 1 mM MgSO_4_ and 5 mM ATP, at 37°C. Total ATPase activity was expressed as nanomoles of Pi per minute per microgram of protein. To determine the contribution to total ATPase activity made by the F_1_F_O_-ATPase, the activity was also measured after adding DCCD (dissolved in ethanol) to a final concentration of 0.2 mM. Subtraction of the activity remaining in the sample after DCCD treatment from the total ATPase activity yielded the contribution made by the F_1_F_o_-ATPase. Protein concentration in the membrane vesicles was determined by the Lowry method using bovine serum albumin as a standard (55).

### Measurement of H_2_ production via gas chromatography

Accumulated H_2_ was measured in the headspace of cultures grown anaerobically for 10 h in 15 mL Hungate tubes in M9-glucose minimal medium (10 mL headspace), initially under a N_2_ atmosphere, and at 37°C (17). Two independent experiments were done, and the amount of H_2_ was calculated with reference to the volume of gas headspace and is presented as an average of the two measurements.

## SUPPLEMENTAL MATERIAL

Supplemental materials jb.00007-26-s0001.pdf Fig. S1 to S4 and additional experimental details.

ASM does not own the copyrights to Supplemental Material that may be linked to, or accessed through, an article. The authors have granted ASM a non-exclusive, world-wide license to publish the Supplemental Material files. Please contact the corresponding author directly for reuse.
