# Supplementary material for: Formate hydrogenlyase is essential for pH homeostasis, maintenance of ATP levels, and CO2 provision in stationary-phase Escherichia coli
Source: J Bacteriol. 2026 May 28;208(6):e00007-26. doi: 10.1128/jb.00007-26 (PMC13277316; doi:10.1128/jb.00007-26)
Supplement: Supplemental materials — Fig. S1 to S4 and additional experimental details. [file jb.00007-26-s0001.pdf]

Supplementary Material

Formate hydrogenlyase is essential for pH homeostasis, maintenance of ATP levels and CO_2 provision in stationary-phase cells of *Escherichia coli*

Christopher Erdmann[a], Liana Vanyan[b], Karen Trchounian[b], and R. Gary Sawers[a]#

[a] Institute for Microbiology, Martin Luther University Halle-Wittenberg, Halle (Saale), Germany

[b] Department of Biochemistry, Microbiology and Biotechnology, Faculty of Biology, Yerevan State University, Yerevan, Armenia

Running head: Stationary phase FHL-1-dependent CO_2 and pH homeostasis

# Address correspondence to R. Gary Sawers, gary.sawers@mikrobiologie.uni-halle.de

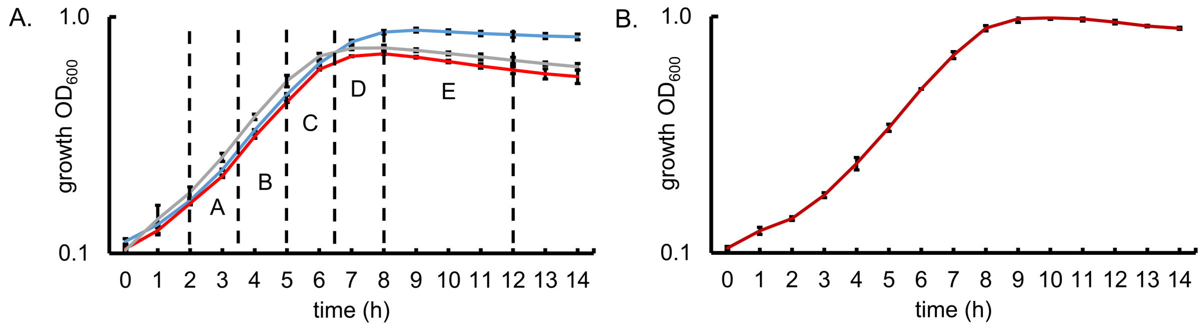

Figure S1. Reduced final OD_{600} attained during glucose fermentation by mutants lacking FHL-1. A. The vertical dotted lines distinguish the very short lag-phase (A), the early (B), mid (C) and late (D) exponential growth phases and stationary phase (E). Strains include the parent, DH4100 (blue line), DH5000 (Δ*fhlA*) (light red line), and HD700 (Δ*hycA-I*) (gray line). B. Growth of DH5000 transformed with plasmid pSA32 (*fhlA*$^+$). The growth experiments were performed with three biological and three technical replicates for each strain. Standard error of the mean is shown for each data point.

Methodology supporting Figure S2 -pH_i determination using the pHrodo Red fluorescent dye

pHi was also determined using pHrodo Red (Thermo Fischer Scientific, USA). Like BCECF-AM, the pH-sensitive group of pHrodo is protected by addition of an ester group, which is then cleaved by non-specific esterases after uptake into the cell. Cell harvest and treatment was done essentially identically to that described for BCECF-AM incubation (see Materials and Methods of main text). Culture samples were centrifuged for 10 min at 9,000 rpm at 4 °C and the cells were suspended and washed in 50 mM HEPES, pH 7.4 (Thermo Fischer) to remove residual medium components. After centrifugation for a further 10 min at 4 °C, the cell pellet was suspended in 200 μl

of the buffer supplied in the kit, to which was added pHrodo fluorescent dye to a final concentration of 5 μM. The samples were then incubated in the dark at 37 °C for 20 min. After a further centrifugation step, the cells for determination of the pHi were suspended in 300 μl of 50 mM TMBT buffer, pH 7.0, while cell samples used to generate the calibration curve were suspended in 240 μl of TMBT buffer prepared across the pH range between 5 and 7.5, to which 60 μl of KCl-CCCP solution were subsequently added (see Materials and Methods in main text). The samples were incubated in the dark at 37 °C for 10 min before measuring the fluorescence at 550 nm (excitation) and 605 nm (emission), which was also performed at 37 °C. Reproducible data (see e.g., Fig. A2 below) using this fluorescent dye could only be obtained for low-pH samples and thus the method was only suitable for use with stationary-phase culture samples.

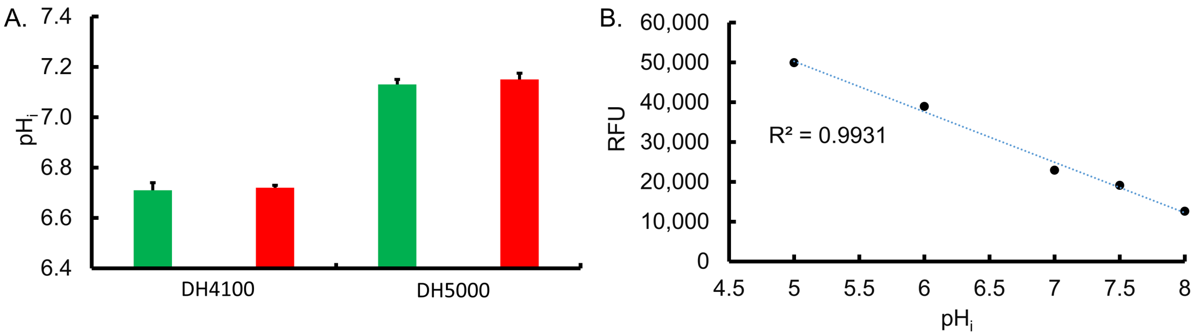

Figure S2. Comparison of methods to determine pH_i in stationary phase cells.

A. The pH_i in cells from stationary phase cultures (phase E) of the parental strain, DH4100, and the *fhlA* mutant, DH5000 was determined using two different fluorescence methods. Green histograms represent pH_i determined using BCECP-AM (see main text for details), while red histograms show pHi determined using pHrodo Red. B. Shown is the calibration curve determined for pHrodo Red at different pH_i (see method above).

57

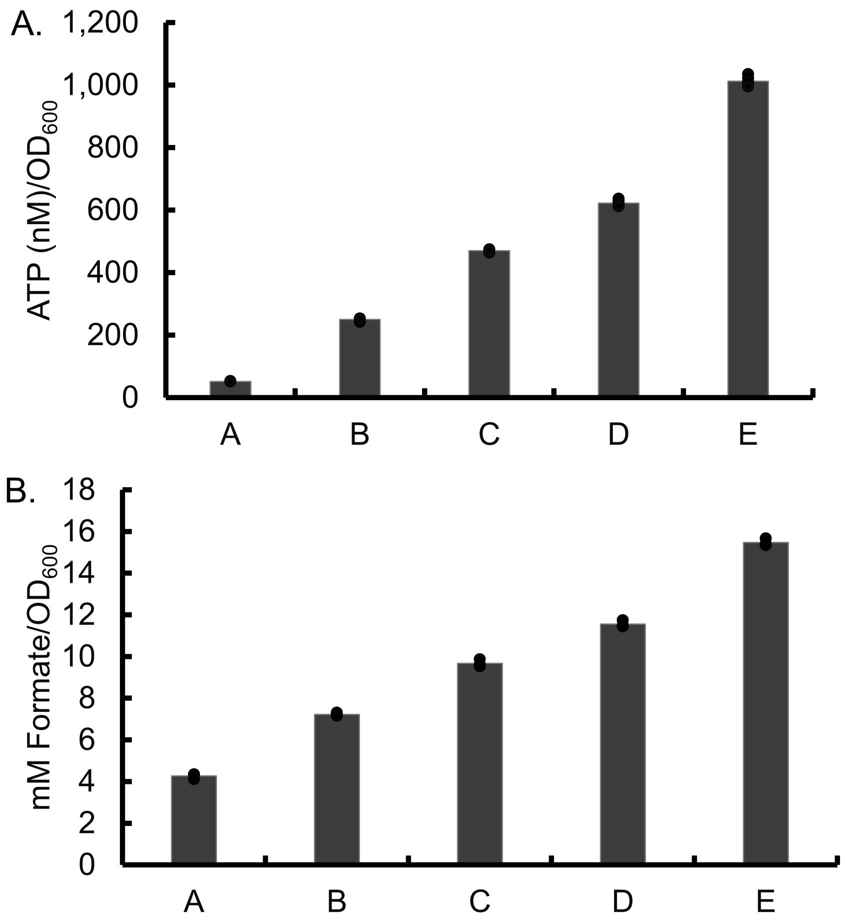

Figure S3. ATP and extracellular formate levels in strain HD700 transformed with pRBH (*hycA-I*$^+$). Data are shown for intracellular ATP levels (part A) and for formate concentration (part B) in the culture medium during growth of HD700/ pRBH. Parameters were determined at phases: A, 3h; B, 4h; C, 6h; D, 8h; E, 10h. Data points are shown for three biological replicates.

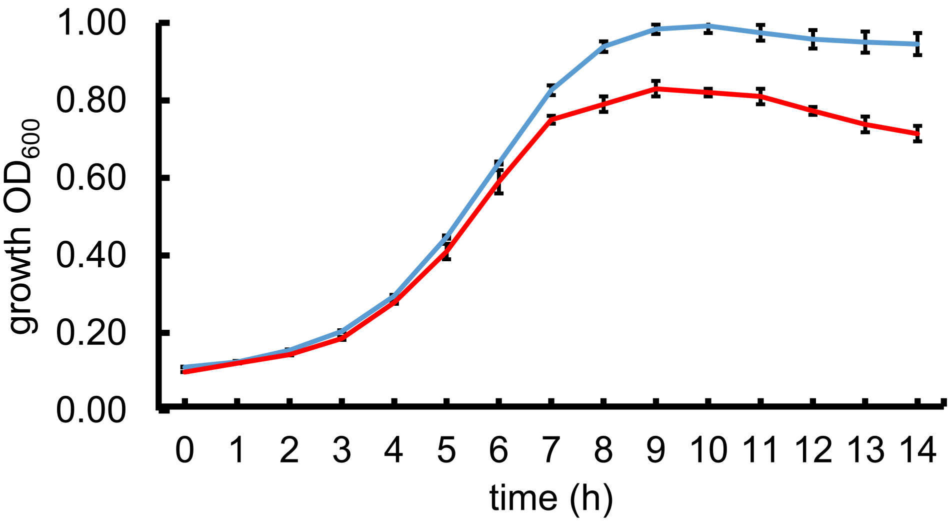

Figure S4. Fermentative growth of DH4100 and DH5000 (Δ*fhlA*) grown in M9-glucose minimal medium, pH 8. Anaerobic growth curves of DH4100 (blue line) and DH5000 (light red line) in M9-glucose, pH 8, are shown. The growth experiment was performed with three biological and three technical replicates for each strain. Standard error of the mean is shown for each data point.
